# Knowledge-attitude and practice of Anthrax and brucellosis: Implications for zoonotic disease surveillance and control in pastoral communities of Afar and Somali region, Ethiopia

**DOI:** 10.1371/journal.pntd.0012067

**Published:** 2024-04-04

**Authors:** Rea Tschopp, Ashenafi GebreGiorgis Kidanu

**Affiliations:** 1 Department of Epidemiology and Public Health, Swiss Tropical and Public Health Institute, Allschwil, Switzerland; 2 University of Basel, Switzerland; 3 One Health Division, Armauer Hansen Research Institute, Addis Ababa, Ethiopia; Colorado State University, UNITED STATES

## Abstract

**Background:**

Anthrax and brucellosis are endemic national priority zoonotic diseases in Ethiopia. This study assess the possible factors explaining the current limited information available on animal and human cases in pastoral communities.

**Methods:**

Two questionnaire surveys gathered data from 509 pastoralists and 51 healthcare providers between February and April 2019 in five districts of Afar and the Somali region (SRS).

**Results:**

Among the 51 healthcare providers, 25 (49%) and 38 (74.5%) had heard of brucellosis, and anthrax, respectively. Of those, only 3 (12%) and 14 (36.8%) knew the symptoms of brucellosis and Anthrax. None of the Health Extension Workers knew any disease symptoms. Healthcare providers recalled two human cases of brucellosis and 39 cases of Anthrax in the last 12 months, based on symptom-based diagnosis. Pastoralists had a moderate level of knowledge about diseases in their animals, with over half (52.4%; n = 267/509) understanding that animals can transmit diseases to people. Overall, 280 out of 508 (55.1%) and 333 out of 507 (65.7%) pastoralists had heard of brucellosis and Anthrax, respectively. Among the latter, 282 (51.3%) knew at least one preventive measure for Anthrax. However, disease knowledge among women was poor. Despite their knowledge, pastoralists engaged in risky unprotected animal handling, animal product consumption/usage as well as husbandry behaviors exposing them to pathogens and favoring the spread of diseases. They identified Anthrax as the most important zoonosis (47.6%) and as one of top three diseases suspected to cause mortality in their livestock. Pastoralists highlighted lack of vaccine coverage, availability and their timely administration. Both, pastoralists and healthcare providers stated the lack of disease awareness and the unavailability of drugs in the market as important challenges. Health facilities lacked protocols and standard operating procedures for managing zoonotic diseases, and did not have access to laboratory confirmation of pathogens.

**Conclusion:**

Our study revealed significant under-reporting of Anthrax and brucellosis, and weak prevention and response in humans, mostly associated with poor disease knowledge of healthcare providers. Ability to respond to animal outbreaks was limited by vaccine and drugs availability, timely vaccine administration and the mobility of pastoralists.

## Author summary

Ethiopia is a high burden country for zoonotic diseases. The study showed that although there are limited official records of human Anthrax and brucellosis cases, healthcare providers and pastoralists in the Afar and Somali region recalled such cases in both animals and humans, thus highlighting under-reporting of diseases. Pastoralists had a moderate level of knowledge for Anthrax and brucellosis and over half knew at least one preventive measure. However, they still engaged in risky behavior exposing them to pathogens. Ability to respond to animal outbreaks was challenged by limited vaccine and drugs availability, timely vaccine administration and the mobility of pastoralists. Disease knowledge was relatively poor among healthcare providers. Particularly the community health professionals-as front liners, had no knowledge about Anthrax and brucellosis. Health facilities lacked protocols and standard operating procedures for managing zoonotic diseases, and did not have access to laboratory confirmation of pathogens. The study showed that in these pastoral regions, Anthrax is under-reported, and that surveillance, prevention and responses in humans and livestock remain inadequate. It highlighted the need for a One-Health and stronger trans-disciplinary approach, leveraging the knowledge and attitudes of pastoralists to enhance disease prevention and control efforts.

## Introduction

Zoonotic diseases are infectious diseases transmitted by vertebrate animals to humans, often involving a wildlife component and accounting for the majority of all emerging infection diseases in humans [1]. Their prevalence is expected to increase due to ongoing socio-ecological changes, primarily driven by human activities.

Zoonotic diseases are characterized by a tripled burden: they affect public and animal health, causing illness and potential mortality in both animals and people, and they have significant socio-economic implications, such as loss of markets, and decreased livestock production, hence posing a threat to individual livelihoods, food security and thus contribute to the perpetuation of poverty [2]. Zoonotic diseases are disproportionately prevalent in middle and low-income countries, where the health sectors face multiple challenges, including limited financial and logistic resources for disease surveillance and control, poverty, ecological crisis, weak governance, political instabilities, and climatic shocks, among others. Despite their significant impact, large-scale prevention and control of many zoonotic diseases remain poor to inexistent [3].

Ethiopia bears the second highest burden of zoonotic diseases in Africa [4,5]. The Ethiopian Government has identified five priority zoonotic diseases, namely rabies, anthrax, brucellosis, echinococcosis and leptospirosis. However, addressing these diseases remains a challenge due to various factors. These include a lack of coordination between public and animal health sectors, inadequate laboratory infrastructure, limited surveillance capabilities, insufficient resources, localized unrests and conflict, and the ongoing COVID-19 pandemic. Anthrax, ranking as the second most concerning zoonotic disease after rabies, is a spore-forming acute bacterial disease, caused by *B*. *anthracis*. It is endemic in Ethiopia, causing sporadic outbreaks throughout the country. A review conducted by Bahiru et al (2016) reported 5197 human and 26737 animal cases occurring between 2009 and 2013, with 86 human fatalities [6]. While Tigray and Amhara regions accounted for the highest burden of Anthrax cases, including PCR confirmed reports, no human cases were officially reported from pastoral areas [6,7]). Brucellosis, caused by Brucella spp, is another bacterial disease of concern in Ethiopia. It can cause widespread abortions in ruminants. In humans, brucellosis can cause adverse events during pregnancies and, if left untreated, may result in long-term cardiac, neurological or immunological complications [8]. Brucellosis is also considered endemic in Ethiopia [9].

Ethiopia is predominantly an agrarian society, with over 80% of its population engaged in livestock rearing livestock for subsistence, with a livestock population exceeding 200 million animals [10]. Particularly pastoralist communities living in the Eastern lowland regions of Afar and Somali, own large livestock herds; they engage in seasonal migration in search of fodder and water, allowing them to adapt to recurrent drought conditions. Livestock serve as vital economic asset for these communities and fulfill daily subsistence needs, such as milk, and meat consumption. The average prevalence of Brucellosis in these regions was reported to be 8.8% in livestock and 41.6% in people [9]. Unfortunately, there is no published primary data on Anthrax in these regions. Secondary data, as reviewed by Bahiru et al (2016), indicated a limited number of animal Anthrax cases and no reported human cases [6]. However, suspected outbreaks are regularly reported within the communities. For instance, in 2000, a suspected Anthrax outbreak occurred in Afar, affecting hundreds of individuals and resulting in human fatalities [11,12]. Unfortunately, due to lack of laboratory capacity, the outbreak was never officially confirmed. Anthrax is categorized as an immediately notifiable disease according to the Government’s regulation outlined in the Public Health Emergency Management Guidelines [12]. Under reporting and under- diagnosis continue to pose however, significant challenges. Recognizing the prevalence of neglected zoonotic diseases in Afar and Somali region, as well as the dearth of epidemiological data, we conducted a research study aimed at assessing the knowledge, attitudes, and practices regarding the prevention and control of zoonotic diseases. Specifically, our focus was on Anthrax and brucellosis among pastoral communities and health care providers. The objective of this study was to identify gaps and existing challenges in zoonotic disease surveillance and control interventions in these remote pastoral areas.

## Methods

### Ethics statement

This study received institutional clearance from the Armauer Hansen Research Institute -AHRI/ALERT Ethics Review Committee (AAERC) (nr. P041-17). Additional permission to conduct the study was obtained from the relevant regional and district health bureaus.

### Study area and study design

**Fig 1:**
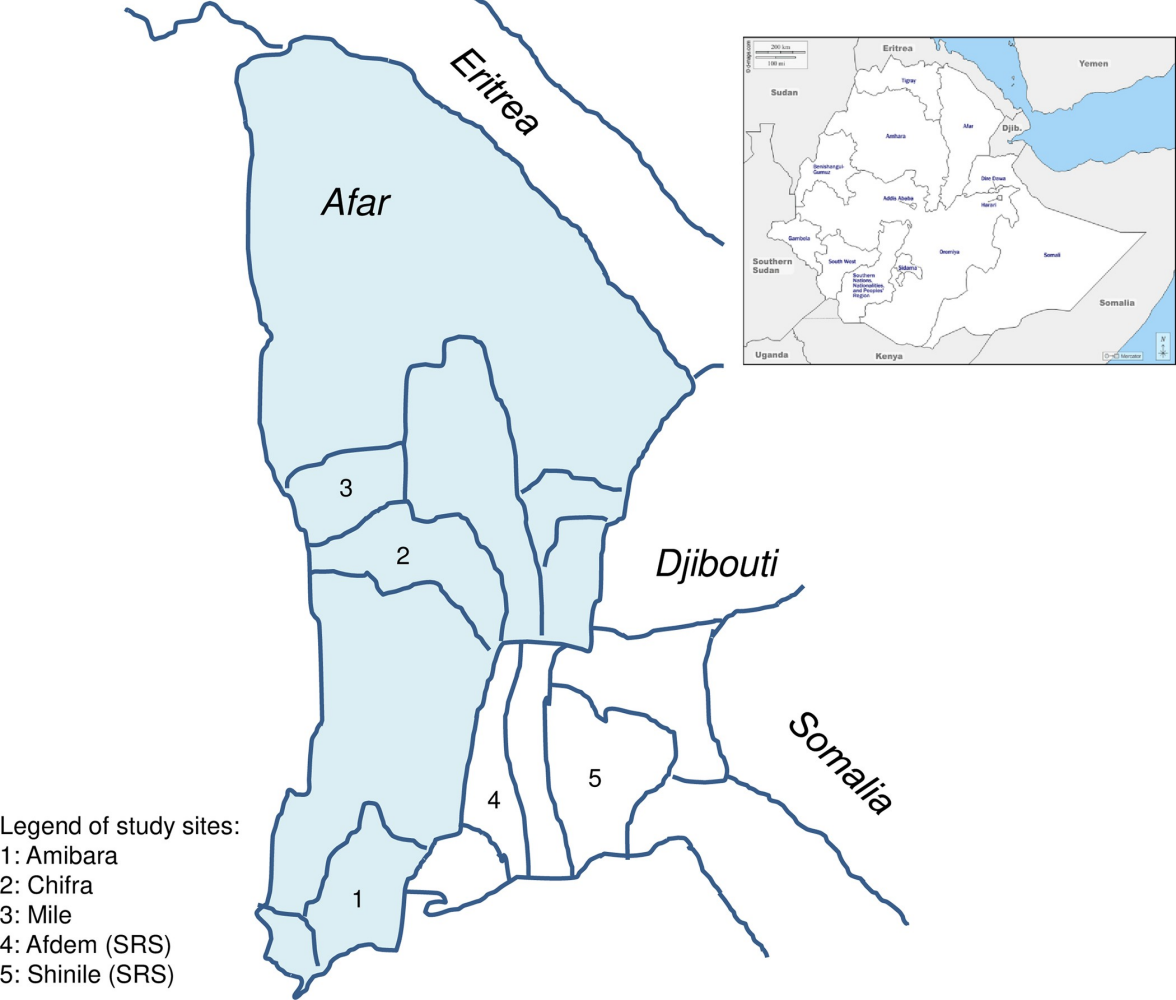
Map of Ethiopia (https://d-maps.com/continent.php?num_con=122&lang=en) and sketch map of the Afar and Northern part of the Somali region showing the selected woredas.

We conducted between February and April 2019, a cross-sectional study survey in the Afar and Somali Regional State (SRS) of Ethiopia. The study included five districts, namely Mile, Chifra and Amibara from Afar, and Shinile and Afdem from SRS (Fig 1). The selection of these districts was based on the presence of existing logistics in the areas by the research team as referred to in Tschopp et al., 2021 [9], security considerations at the time of the study, accessibility to the areas, and the willingness of elders and head of households to participate in the study. These criteria were essential for ensuring the smooth implementation of the research and the cooperation of the local communities.

Sample size calculation was based on the total number of households in the woredas, an estimated KAP positive response of 50%, a 95% confidence level and a margin of error of 5%, thus resulting in total in 384 interviews [13]. We also anticipated that choosing a similar group of pastoral respondents, most people would provide similar answers. However, we increased the sample size according to budget availability. A list of villages (kebeles) was provided by the local authorities, of which villages were randomly selected. Within the selected kebeles, a non-probability sampling was performed (“random walk”). The starting point along the walking path was determined randomly. Every fifth household along the path was then selected until the target number per kebele was reached. Interviewers went physically from house to house.

### Study population and study tools

We conducted two distinct surveys using questionnaires as our data collection method. The first survey targeted pastoral communities in the selected villages. Eligible participants were pastoralists aged 18 years or older who have been living in the study sites for at least one year. To be included in the study, participants needed to be mentally competent and provide written informed consent. The second survey was conducted among health staff working at health facilities and at local health bureaus in each of the selected districts. They were informed about the project and provided signed consent before participating in the survey. Both structured questionnaires included closed and open-ended questions. They were prepared in English, translated into the local languages (Afarinia and Somali) and back-translated into English for validation. They were pre-tested prior the start of the study to ensure that all questions were well understood. Questions were then corrected if needed. A veterinary public health senior researcher administered all the interviews, supported by local translators. This allowed for consistency and standardizing administration process and minimizing interviewer related bias.

The questionnaire utilized in the community survey aimed to gather following information: demographic data, husbandry practices, knowledge of diseases (including causes, transmission, clinical symptoms, prevention and control); attitudes and practices related to zoonotic diseases (such as meat consumption, slaughtering and handling of dead animals, and treatment seeking behaviors). It also included suggestions from the community on how to improve disease surveillance and control based on their perspectives.

The health staff questionnaire collected information on disease knowledge, attitudes, and practices among health care providers. It also sought to identify healthcare challenges and gaps in relation to zoonotic diseases in general, with a specific focus on Anthrax. In addition, the questionnaire gathered suggestions from health staff on how to improve diagnostics and treatment approaches for patients affected by zoonotic diseases, and specifically by Anthrax and brucellosis.

### Data management and analysis

Data was entered into Microsoft Access tables and analyzed descriptively using STATA version 16.1 (StataCorp, USA). Chi square test was used to compare groups.

Knowledge parameters, such as knowledge of disease symptoms, transmission routes and preventive measures were analyzed using a scoring system. A score 0 was assigned to a participant who could not provide any information on the parameters. A score of 1 represented “poor knowledge”, with only one response provided to the selected parameter. Moderate knowledge (score 2) represented up to 50% of right answers being provided. A participant had “good knowledge” (score 3) when he could provide more than 50% of right answers to the parameters. Open-ended questions with qualitative data were analyzed descriptively thematically. A univariate logistic regression was performed for pastoralist respondents to assess variables associated with knowledge. Variables were divided into a group that had no knowledge (score 0) and a group that showed at least one right answer (score 1 to 3). A p-value lower than 0.05 was considered statistically significant.

## Results

### Demography

#### Health care providers

Overall, 51 healthcare providers were interviewed (S1 Data), 28 in Afar and 23 in SRS. The majority were male (70%). The participants included 25 nurses (49%; 25/51), 12 Health Extension workers (HEW) (23.5%;12/51), nine physicians (17.6%; 9/51), three health technicians (5.9%; 3/51) and two health administration staff (3.2%; 2/51). The majority of interviewees were employed in primary health facilities, such as health centers (39.2%; 20/51) and health posts (31.4%; 16/51), followed by private clinics (17.6%; 9/51) and government hospitals (11.8%; 6/51). The majority (64.5%; 29/45) had been employed for less than 5 years and nearly a third (31.1%; 14/45) for less than 2 years. Only six participants (13.3%; 6/45) had been employed for over 10 years at the time of the interview.

#### Pastoralists

Overall, 509 pastoralists participated in the study, 300 from Afar (59%) and 209 from SRS (41%) (S2 Data). The majority of respondents were male (70.3%; 358/509). Interviewees’ age ranged from 19 to 70 years old, with a mean age of 39 years (95%CI: 36–40). Family size ranged from one to 19 with a mean six people (95%CI: 6–7). The majority of respondents were illiterate (74.3%; 378/509), while 23.4% (119/509) and 2.3% (12/509) attended primary and secondary school, respectively. All kept livestock. Overall, 51.4% (261/508) were pastoralists and 48.6% (247/508) agro-pastoralists. In SRS, pastoralists made up 70.8% (148/209) of the respondents. The majority (73.9%; 376/509) engaged in seasonal migration with their animals.

### Disease knowledge

#### Health care providers

Out of the total 51 participants, 25 (49%) had heard about brucellosis, while 38 (74.5%) were familiar with Anthrax. Three individuals (two physicians and one nurse) could accurately describe the symptoms of brucellosis, whereas 14 health staff (five physicians, eight nurses, and two administrative staff) were able to described symptoms related to Anthrax. Only those health care providers who had previously encountered cases of these diseases were knowledgeable about the symptoms. The other interviewees were unable to list any specific symptoms for either disease. Overall, only 55.5% (n = 5) of the nine physicians had knowledge of Anthrax symptoms, while only 22.2% (N = 2) were aware of brucellosis symptoms.

Among the 25 nurses, 32% (n = 8) knew Anthrax symptoms, and only 4% (n = 1) were familiar with brucellosis symptoms. None of the health technicians or HEWs had knowledge of any symptoms for either diseases. Regarding the transmission route to people, 21 out of the 51 participants (41.2%) were aware of the transmission route for brucellosis. This group consisted of all the physicians (100%), 40% of the nurses (n = 10), and both health administrators (100%). However, none of the health technicians or HEWs knew about the transmission routes for these diseases.

Similarly, 35 out of 51 participants (68.6%) claimed to have knowledge of the transmission routes for Anthrax. Among them were seven physicians (77.8%), 22 nurses (88%), both health administrators (100%), one health technician (33.3%), and three HEW (25%). However, a third of the respondents who claimed knowledge of Anthrax transmission did not possess further details. Detailed information on provided disease symptoms and transmission routes are shown in Table 1. Table 2 shows scored knowledge of disease among the interviewed healthcare staff.

**Table 1:** Crude knowledge of disease symptoms and transmission among 51 health staff from Afar and Somali region of Ethiopia stating to know brucellosis and/or Anthrax.

Category	Disease	Number (%)	Sub-categories	Number (%)
Heard about the disease	Brucellosis	25 (49.0)		
	Anthrax	38 (74.5)		
Symptoms	Brucellosis	3 (12.0)	Joint pains	3 (100)
			Testes swelling/pain	3 (100)
			Extreme sweating	1 (33.0)
			Fatigue	1 (33.0)
			Intermittent fever	1 (33.0)
	Anthrax	14 (36.8)	Vomiting blood	9 (64.3)
			Skin lesions*	8 (57.2)
			Chest pain/painful breathing	7 (50.0)
			Fever	5 (35.7)
			Difficulty breathing	5 (35.7)
			Coughing blood	4 (28.6)
			Abdominal pain	4 (28.6)
			Bloody diarrhea	4 (28.6)
			Oral froth	3 (21.4)
			Swelling of the face	3 (21.4)
			Nausea	2 (14.3)
			Inflamed conjunctivae	2 (14.3)
			Pain when swallowing	2 (14.3)
			Sweating	2 (14.3)
			No clotting of the blood	1 (7.1)
			Headaches	1 (7.1)
			Swollen abdomen	1 (7.1)
Transmission	Brucellosis	21 (84.0)	Unprotected contact with aborted material/vaginal discharge	17 (80.9)
			Raw milk consumption	10 (47.6)
			Contact with animals-unspecific	5 (23.8)
	Anthrax	35 (92.1)	Contact with sick animal discharge incl. unclotted blood	18 (51.4)
			Consumption of meat/organs from infected animal	12 (34.3)
			Contact with sick animals-unspecific	11 (31.4)
			Upon opening carcasses of infected animals	5 (14.3)
			Aerosol transmission(inhalation of spores	5 (14.3)
			Consumption of raw milk	4 (11.4)
			Skin of infected animal	1 (2.9)

*skin lesions: half of these respondents stated a typical black center

**Table 2:** Score based knowledge of health personnel (N = 51) and pastoralists (N = 509) interviewed in the pastoral regions of Afar and Somali, Ethiopia (disease symptoms, transmission and preventive measures) towards Anthrax and brucellosis.

		Health staff		Pastoralists	
Category	Scoring	Brucellosis	Anthrax	Brucellosis	Anthrax
		Number (%)	Number (%)	Number (%)	Number (%)
Symptoms in people	No knowledge (score 0)	48 (94.1)	37 (72.5)	229 (45.0)	262 (51.5)
	Poor knowledge (score 1)	0	1 (2.0)	174 (34.2)	50 (9.8)
	Moderate knowledge (score 2)	3 (5.9)	13 (25.5)	65 (12.8)	197 (38.7)
	Good knowledge (score 3)	0	0	41 (8.0)	0 (0)
Disease transmission to people	No knowledge (score 0)	30 (58.8)	19 (37.5)	449 (88.2)	264 (51.9)
	Poor knowledge (score 1)	17 (33.3)	25 (49.0)	60 (11.8)	197 (38.7)
	Moderate knowledge (score 2)	3 (5.9)	3 (5.9)	0 (0)	48 (9.4)
	Good knowledge (score 3)	1 (2.0)	4 (7.8)	0 (0)	0 (0)
Preventive measures in people	No knowledge (score 0)	34 (66.7)	21 (41.2)	449 (88.2)	263 (51.7)
	Poor knowledge (score 1)	13 (25.5)	13 (25.5)	60 (11.8)	189 (37.1)
	Moderate knowledge (score 2)	3 (5.9)	8 (15.7)	0 (0)	51 (10.0)
	Good knowledge (score 3)	1 (2.0)	9 (17.6)	0 (0)	6 (1.2)
Symptoms in animals	No knowledge (score 0)	-	-	-	178 (35.0)
	Poor knowledge (score 1)	-	-	-	127 (25.0)
	Moderate knowledge (score 2)	-	-	-	192 (37.7)
	Good knowledge (score 3)	-	-	-	12 (2.3)
Livestock preventive measures	No knowledge (score 0)	-	-	-	248 (48.7)
	Poor knowledge (score 1)	-	-	-	191 (37.5)
	Moderate knowledge (score 2)	-	-	-	57 (11.2)
	Good knowledge (score 3)	-	-	-	13 (2.6)

#### Community level

More than half of the 509 respondents (n = 267; 52.4%) reported that animals can transmit diseases to people, whereas 6.7% (n = 34) thought this to be impossible and 40.9% (n = 208) did not know. Among those 267 people who had knowledge, the most frequently reported transmission routes from animals to people was the consumption of animal products originated from sick animals (58.8%; n = 157)). The second transmission route mostly reported was contact with lesions or discharges from sick animals (29.6%; n = 79) or generally being in close contact with a sick animal (24.3%; n = 65). Less frequently reported routes included bites wounds (12%; n = 32), aerosol transmission (10.9%; n = 29), insect bites (0.4%; n = 1) and contaminated water or food (0.4%; n = 1). Two people (0.8%) did not know any transmission routes.

The top four zoonotic diseases reported by the 267 pastoralists who had knowledge about zoonotic diseases, were Anthrax (n = 127; 47.6%), Tuberculosis (n = 86; 32.2%), Foot and Mouth disease (n = 22; 8.2%) and rabies (n = 18; 6.7%).

Overall, 25% (126/504) of the interviewed households reported disease outbreaks in the last 12 months. These were mainly outbreaks of Peste des Petits Ruminants (PPR), Pox Virus, pasteurellosis and Anthrax. Over half of the respondents (59.2%; 298/503) claimed that some of their animals died either during large-scale disease outbreaks or due to singular diseases. The top three causes of mortality affecting the different livestock species as reported by the pastoralists are shown in Table 3.

**Table 3:** Top three diseases listed by pastoralists in Afar and Somali region (N = 509) causing death in their different livestock species.

Livestock species	Afar			SRS		
	Total HH	Diseases	Nb HH (%)	Total HH	Diseases	Nb HH (%)
Cattle	109	CBPP	33 (30.3)	35	LSD	10 (28.6)
		Pasteurellosis	32 (29.3)		**Anthrax**	8(22.8)
		**Anthrax**	22 (20.2)		Trypanosomosis	6 (17.1)
Camel	69	Pasteurellosis	19 (27.5)	28	Chronic Respiratory Disease Complex	22 (78.6)
		Chronic Respiratory Disease Complex	17 (24.6)		Trypanosomosis	4 (14.3)
		Trypanosomosis	14 (20.3)		**Anthrax**	2 (7.1)
Goats	191	Pasteurellosis	83 (43.4)	85	Pox	49 (57.6)
		PPR	83 (43.4)		PPR	23 (27.0)
		Pox	38 (19.9)		Pasteurellosis	21 (24.7)
Sheep	113	Pasteurellosis	71 (62.8)	86	Pox	52 (60.5)
		Pox	31 (27.4)		Pasteurellosis	25 (29.0)
		PPR	25 (22.1)		PPR	19 (22.0)

Respondents reported acquiring information about zoonotic diseases from various sources. The majority of the 267 respondents (65.9%; n = 176) obtained knowledge from elders or members of their community, while 36.3% (n = 97) received information from animal health professionals, and 28.1% (n = 75) learned from their own family members. Mass media sources, such as television, radio, or newspapers, were cited by 10.9% (n = 29) of the interviewees as a means of gaining knowledge about these diseases. Health extension workers (4.1%; n = 11) and religious leaders (4.9%; n = 13) played a minor role in zoonotic disease knowledge transfer.

Overall, 280 out of 508 participants (55.1%) had heard of brucellosis (81.3%; n = 244/300 of the Afar respondents and 17.3%, n = 36/208 of the SRS respondents). Among them, all stated abortions in animals as a main disease symptom. A quarter (25.3%; n = 71) also mentioned retained fetal/uterine membranes, 19.6% (n = 55) stillbirth and 3.9% (n = 11) smelly uterine discharge. The majority (78.1%; n = 239) did not know how brucellosis is transmitted or thought it was not transmitted at all from animals to humans. The other 21.9% mentioned following transmission routes: contact with sick animals (n = 24, 8.6%), contact with aborted material (n = 23; 8.2%) and consumption of products from sick animals (n = 13; 4.6%). The existence of a therapy for brucellosis in livestock and people was unbeknown by 48.1% (n = 134) and 95% (n = 265) of these 280 respondents, respectively.

Overall, 333 out of 507 respondents (65.7%) stated to have knowledge about Anthrax. Of those, 113 (33.9%) did not know how it is transmitted, and 68 (20.4%) did not know it was a zoonotic disease. The majority (84.7%; n = 282) knew at least one preventive measure. A quarter of the pastoralists (82/329; 25.9%) did not know that livestock can be treated for Anthrax. Furthermore, 147 out of 324 interviewees (45.4%) did not know people could be treated for Anthrax. Table 2 shows scored knowledge among pastoralists towards brucellosis and Anthrax.

Univariate logistic regression analysis of knowledge of disease symptoms, transmission and their prevention is shown in Table 4. The analysis highlights that region, sex, education level and whether pastoralists are migrating are all variables which have statistical significance in disease knowledge.

**Table 4:** Univariate logistic regression analysis of knowledge of disease symptoms, transmission and prevention among the 509 interviewed pastoralists in Afar and Somali region, Ethiopia.

				Brucellosis			Animal Anthrax			Human Anthrax		
	Variables	Group	Total number	N (%)	OR (95%CI)	p-value	N (%)	OR (95%CI)	p-value	N (%)	OR (95%CI)	p-value
Knowledge of symptoms	Region	Afar	300	244 (81.3)	-		222 (74.0)	-		157 (52.3)	-	
		SRS	209	36 (17.2)	0.04 (0.03–0.07)	<0.001	110 (52.6)	0.4 (0.3–0.6)	<0.001	90 (43.0)	0.6 (0.5–0.9)	0.04
	Sex	Female	151	47 (31.1)	-		58 (38.4)	-		33 (21.8)	-	
		Male	358	233 (65.1)	4.1 (2.7–6.2)	<0.001	274 (76.5)	5.2 (3.4–7.8)	0.005	214 (59.8)	5.3 (3.4–8.2)	<0.001
	Education	Illiterate	378	238 (63.0)	-		294 (77.8)	-		237 (62.7)	-	
		Primary	119	37 (31.1)	0.3 (0.2–0.4)	<0.001	35 (29.4)	0.1 (0.07–0.2)	<0.001	10 (8.4)	0.05 (0.02–0.1)	<0.001
		Secundary	12	5 (41.7)	0.4 (0.1–1.3)	0.14	3 (25.0)	0.09 (0.02–0.3)	0.001	0 (0)	-	
	Migration	No	133	42 (31.6)	-		33 (24.8)	-		14 (10.5)	-	
		Yes	376	238 (63.3)	3.7 (2.4–5.7)	<0.001	299 (79.5)	11.8 (7.4–18.7)	<0.001	233 (61.7)	13.8 (7.7–25.0)	<0.001
Knowledge of transmission	Region	Afar	300	62 (20.7)	-					155 (51.7)	-	
		SRS	209	0 (0)	-					90 (43.0)	0.7 (0.5–1.0)	0.06
	Sex	Female	151	12 (7.9)	-					32 (21.2)	-	
		Male	358	50 (14.0)	1.9 (0.9–3.6)	0.06				213 (59.5)	5.4 (3.5–8.5)	<0.001
	Education	Illiterate	378	57 (15.0)	-					236 (62.4)	-	
		Primary	119	5 (4.2)	0.2 (0.09–0.6)	0.003				9 (7.5)	0.04 (0.02–0.1)	<0.001
		Secundary	12	0 (0)	-					0 (0)	-	
	Migration	No	133	5 (3.8)	-					16 (12.0)	-	
		Yes	376	57 (15.1)	4.5 (1.8–11.6)	0.002				229 (60.9)	11.4 (6.5–19.9)	<0.001
Knowledge of prevention	Region	Afar	300	60 (20.0)	-		173 (57.7)	0.5 (0.4–0.7)	0.001	156 (52.0)	0.7 (0.5–1.0)	0.04
		SRS	209	0 (0)	-		88 (42.1)			90 (43.0)		
	Sex	Female	151	12 (7.9)	-		36 (23.8)	-		34 (22.5)	-	
		Male	358	50 (14.0)	1.8 (0.9–3.5)	0.08	225 (62.8)	5.4 (3.5–8.3)	<0.001	212 (59.2)	5.0 (3.2–7.7)	<0.001
	Education	Illiterate	378	56 (14.8)	-		241 (63.7)	-		237 (62.7)	-	
		Primary	119	4 (3.4)	0.2 (0.07–0.5)		19 (16.0)	0.1 (0.06–0.2)	<0.001	9 (7.6)	0.04 (0.02–0.09)	<0.001
		Secundary	12	0 (0)	-		1 (8.3)	0.05 (0.006–0.4)	0.005	0 (0)	-	
	Migration	No	133	5 (3.7)	-		21 (15.8)	-		15 (11.3)	-	
		Yes	376	5 (1.3)	4.4 (1.7–11.2)	0.002	240 (63.8)	9.4 (5.6–15.7)	<0.001	231 (61.4)	12.5 (7.0–22.3)	<0.001

### Attitude and practice

#### Health care providers

Overall, two (3.9%) and 14 (27.4%) out of the 51 respondents had during their career encountered cases of brucellosis and Anthrax, respectively. Within the 12 months preceding the interviews, participants reported two cases of human brucellosis, with one occurring in Afar and one in SRS. Additionally, there were 39 cases that were treated as suspected human Anthrax, with 17 cases in Afar and 22 cases in SRS. Among the 14 health care providers stating having encountered Anthrax cases, 10 reported them in adults (71.4%), while 4 (28.6%) involved both adults and children. Cases were described as sporadic, with the number of reported cases ranging from zero to five per year.

#### Community

The survey assessed potential risks associated with zoonotic diseases. Overall, 8% (32/388) of the respondents reported actively avoiding specific grazing areas, even during migration. The reasons provided for this avoidance included the lack of water, stagnant water or swampy areas that were prone to a high burden of liver fluke, areas known for high tick prevalence and locations forbidden by elders or legally unauthorized, such as National parks. Almost all respondents (99.2%; n = 504/508) reported providing regular assistance during animal birth, and with the exception of one person, all participants reported handling animal afterbirths with their bare hands. Furthermore. A significant majority (99.4%) reported regularly consuming raw milk. Small ruminants were slaughtered in the backyard of 90.9% of the households (n = 462/508). Additionally, 59.5% (n = 292/491) and 57% (n = 280/490) of the interviewees reported slaughtering cattle and camels at home, respectively. Overall, muscle meat was generally consumed cooked (99%; n = 504/509). However, a third (35.1%; n = 179/509) of the respondents consumed raw animal organ meat.

Diseased animals were home-slaughtered in 20.7% (n = 105/508) of the households and their meat also consumed- albeit respondents stated that the meat was always cooked before consumption. Nearly all households who used parts of slaughtered sick animals (98.1%; n = 52/53) utilized hides and wool from diseased animals.

### Disease diagnostic, prevention and treatment

#### Health care providers

All respondents who were knowledgeable about Brucellosis stated that cases were diagnosed based on symptoms alone. In the case of Anthrax, six out of a total of 33 individuals who knew Anthrax (18.2%) mentioned that they would seek laboratory confirmation for diagnosis, while 27 individuals (81.8%) relied solely on symptom-based diagnosing.

Out of the 14 healthcare providers who were familiar with brucellosis, four (28.6%) stated that they would treat the symptoms, while two (14.3%) mentioned attempting treatment with antibiotics. However, seven respondents (50%) expressed their inability to treat the disease due to the unavailability of drugs in the market, and five respondents stated that their facility was not equipped to handle such cases, thus preventing them from providing treatment.

Among the 34 respondents with knowledge of Anthrax, the primary treatment approach varied. First aid was mentioned as the initial step by 10 individuals (29.4%), followed by referring the cases to a better-equipped facility, as reported by seven respondents (20.6%). In the Somali Regional State (SRS), Anthrax cases were referred to the Dire Dawa General Hospital. Eight health care providers (23.5%) stated they would prescribe antibiotics if the drugs were available on the market. However, five people (14.7%) reported the unavailability of drugs as a limitation in providing treatment. Three interviewees (8.8%) mentioned dressing the skin lesions as part of the treatment approach. Five respondents (14.7%) admitted to not know how to treat Anthrax. The remaining 17 respondents, including all HEWs and health technicians, did not suggest any specific treatment options as they lacked knowledge about the disease.

#### Community

Preventive measures during a disease outbreak (with particular emphasis on Anthrax) undertaken by the owners, the community and the animal health workers-from a pastoralists perspective are shown in Table 5. Owners primarily implemented two main actions: separating sick animals from healthy ones (35.4%; n = 115) and avoiding contact with sick animals and their discharge (31.1%; n = 101). The community at large reported cases to their village leaders and/or local authorities (25.9%; n = 84) and attempted to vaccinate their animals (32.7%; n = 106). Animal health workers were perceived to play a crucial role by treating sick animals (55%; n = 176), vaccinating healthy animals (52.5%; n = 168), and raising awareness within the community (20.6%; n = 66).

**Table 5:** Afar and Somali pastoralists point of view of the perceived role of stakeholders in Anthrax outbreak response.

Preventive measures	By individual owners (N = 325)	By the community (N = 324)	By the animal health professionals (N = 320)
Don’t know	34 (10.5)	30 (9.2)	13 (4.0)
Reporting to local authorities	51 (15.7)	84 (25.9)	
Burning carcasses	29 (8.9)	5 (1.5)	
Burrying carcasses	14 (4.3)	2 (0.6)	
Separating sick animals	115 (35.4)	27 (8.3)	
Avoid touching dead animal/opening carcasses	21 (6.5)		
Avoid contact with sick animals and its discharges	101 (31.1)	17 (5.2)	
Avoid grazing in contaminated area	9 (2.8)	15 (4.6)	
Move animals to other areas	39 (12.0)	58 (17.9)	
Avoid consumption of sick animal products	54 (16.6)	13 (4.0)	
Treat sick animals	8 (2.5)	17 (5.2)	176 (55.0)
Vaccinate healthy animals	56 (17.2)	106 (32.7)	168 (52.5)
Avoid slaughtering sick animals	25 (7.7)	2 (0.6)	
Eliminate sick animal	1 (0.3)		
Praying	6 (1.8)	11 (3.4)	
Provision of clean fodder	1 (0.3)		
Tell other people about cases		14 (4.3)	
Help each other with carcass removal (burry/burn)		7 (2.1)	
Provide community awareness (total), include the below			66 (20.6)
* Tell people to move animals to other places*			9
* Tell people not to open carcasses*			5
* Separate infected herds*			4
* Burn carcasses*			2
* Organize community to work together to destroy dead animals*			5
* Tell people not to eat products*			2
*Provision of drugs to the community*			12 (3.7)
*Report back to authorities to get vaccines*			12 (3.7)
Nothing (total), incl below	3 (0.9)	3 (0.9)	18 (5.6)
* Nothing*, *it comes from God*			2
* Nothing because disease fast and fatal*			6
* Nothing*			10

Overall, less than half of the respondents (46.9%; n = 237) had vaccinated their animals in the last 12 months and over a third (37.9%; n = 155) reported vaccinating their animals only after an outbreak has been declared, rather than as a prevention (Table 6).

**Table 6:** Information on animal vaccination as perceived by Afar and Somali pastoralist respondents.

	Category	Overall	Afar	SRS
Last vaccination (n = 505 overall; n = 297 Afar; n = 208 SRS)	This year	237 (46.9)	138 (46.5)	99 (47.6)
	Last year	68 (1.5)	30 (10.1)	38 (18.3)
	2 years ago	110 (21.8)	72 (24.2)	38 (18.3)
	More than 2 years ago	20 (4.0)	19 (6.4)	1 (0.5)
	Never	34 (6.7)	24 (8.0)	10 (4.8)
When are animals vaccinated? (n = 409 overal, n = 247 Afar; n = 162 SRS)	As prevention	272 (66.5)	144 (58.3)	128 (79.0)
	Only during outbreaks	155 (37.9)	109 (44.1)	46 (28.4)
Who performs vaccinations? (n = 404 overall; n = 247 Afar; n = 157 SRS)	Community Animal Health Worker (CAHW)	269 (66.6)	123 (49.8)	146 (93.0)
	District veterinary technician	365 (90.3)	241 (97.6)	124 (85.3)
	Myself	1 (0.2)	1 (0.4)	0 (0.0)
	A friend	1 (0.2)	1 (0.4)	0 (0.0)
	I don t know	2 (0.4)	0 (0.0)	2 (1.3)
Source for vaccines (n = 401 overall, n = 245 Afar; n = 156 SRS)	Woreda	325 (81.0)	208 (84.9)	117 (75.0)
	Kebele	74 (18.5)	35 (14.3)	39 (25.0)
	Private veterinary clinic	2 (0.5)	2 (0.8)	0 (0.0)
	Market	2 (0.5)	2 (0.8)	0 (0.0)
	Drug shop	0 (0.0)	0 (0.0)	0 (0.0)
Why do you think it is important to vaccinate animals? (n = 507 overall, n = 298 Afar; n = 209 SRS)	To protect the animals	385 (75.9)	234 (78.5)	151 (72.2)
	To treat sick animals	61 (12.0)	28 (9.4)	33 (15.8)
	Vaccines are not good for animals, makes them sick	20 (3.9)	13 (4.3)	7 (3.3)
	To improve the animal’s body condition	1 (0.2)	1 (0.3)	0 (0.0)
	It is not important to vaccinate	1 (0.2)	0 (0.0)	1 (0.5)
	Don’t know	39 (7.7)	22 (7.4)	17 (8.1)

Among the 33 pastoralists who never vaccinated their animals, 24 (72.7%) claimed it would make their animals sick, and nine (27.3%) reported that there was no Government vaccination service in their area. Overall, 71.8% of the pastoralists knew the type of vaccines that were provided to their animals. These included vaccination against Lumpy Skin Disease (LSD), pasteurellosis, Contagious Bovine Pleuropneumonia (CBPP), Anthrax, Pox, PPR and Foot and Mouth Disease (FMD). Most pastoralists raised concerns regarding vaccination (Table 7). The major challenge reported (44.2%; n = 224) was the shortage of available vaccines.

**Table 7:** Perceived challenges regarding vaccines as stated by Afar and Somali pastoral respondents in Ethiopia (N = 507).

Categories	Number (%)	Remark
Shortage of vaccines	224 (44.2)	Particularly for large animals such as camels (N = 98)
Lack of animal health service	163 (32.1)	i.e. poorly qualified staff, animal health facilities closed or too far away
Lack of regular preventive vaccination campaigns	162 (31.9)	
Delayed vaccination	93 (18.3)	Animal health workers come only when the outbreak is already well on its way or when people have moved to another place during outbreaks
Lack of vaccine variety	64 (12.6)	
Poor vaccine quality	42 (8.3)	Ineffective or harmful (makes animal sick)
Lack of surveillance	24 (4.7)	Hence no vaccination
Lack of awareness in communities regarding vaccines	5 (1.0)	
No challenges	37 (7.3)	
Don’t know	17 (3.3)	

Overall, when sick, household members would go to nearby health facilities (86.0%; n = 437/508) and 14.4% (n = 73/508) would go to traditional healers. Traditional healers were predominantly visited in SRS (26.3%; 55/209) as compared to Afar (6%; 18/299). A further 0.8% (n = 4/508) would self-medicate and one person said they would do nothing about it.

### Reported challenges and suggestions

#### Health care providers

Table 8 shows the challenges reported by health care providers and their suggestions in order to improve surveillance and control of zoonotic diseases, such as Anthrax and brucellosis.

**Table 8:** Reported challenges by interviewed health care providers in Afar and Somali regions in Ethiopia and their suggestions for Anthrax and Brucellosis surveillance and control.

	Categories	Brucellosis	Anthrax
Reported challenges (brucellosis n = 24; Anthrax n = 23)	Lack of awareness among health staff	20 (83.3)	14 (60.9)
	Lack of laboratory confirmation	4 (16.7)	6 (26.1)
	Lack of preparedness/lack of protocols for zoonosis	2 (8.3)	1 (4.3)
	Lack of community awareness	1 (4.2)	4 (17.4)
	Lack of clear patient history	1 (4.2)	1 (4.3)
	Facilities not equipped for zoonosis	-	4 (17.4)
	Patients are coming too late	-	3 (13.0)
	Lack of available medications	-	2 (8.7)
	Patients refuse to be separated from families	-	2 (8.7)
	Lack of coordination between public and animal health sectors	-	1 (4.3)
Suggested measures for improvement (brucellosis n = 18, Anthrax n = 28)	Increase health staff awareness	15 (83.3)	8 (28.6)
	Increase community awareness	9 (50.0)	16 (57.1)
	Improve laboratory diagnostics	3 (16.7)	4 (14.3)
	Improve facilities to accommodate zoonotic cases	2 (11.1)	6 (21.4)
	Improve reporting system to sector bureaus	1 (5.6)	1 (3.6)
	Avoid consuming raw animal products (milk/meat)	1 (5.6)	1 (3.6)
	Have protocol in place for zoonosis	1 (5.6)	1 (3.6)
	Avoid backyard slaughtering of sick animals	-	3 (10.7)
	Capacitate animal health professionals incl. animal vaccination	-	4 (14.2)
	Provision of drugs and lab supplies	-	1 (3.6)
	Improve trust/cooperation between patient and health care provider	-	1 (3.6)
	Avoid contact with animals	-	1 (3.6)

#### Community

Table 9 shows strategies suggested by Afar and Somali pastoralist respondents in order to improve the prevention of future Anthrax outbreaks. They also provide suggestions on the various challenges for the implementation of preventive and control strategies.

**Table 9:** Strategies suggested by Afar and Somali pastoralists in Ethiopia to improve prevention of future Anthrax outbreaks and challenges for implementation.

Categories	Suggested strategies (N = 326)	Number (%)
No opinion		76 (23.3)
Pastures, fodder, water	Moving animals to other good pastures during dry season	23 (7.0)
	Provision of additional fodder during dry season	3 (0.9)
	Avoid animals to graze on known contaminated pastures	3 (0.9)
	Refrain animals from licking soil	2 (0.6)
	Refrain animals from drinking hot water	1 (0.3)
	Provision of water during dry season	1 (0.3)
	Never graze in thorny feeding area	1 (0.3)
	Avoid wildlife on pastures	1 (0.3)
Migration	Avoid migrating to areas with anthrax history	1(0.3)
Prayers	Praying it does not happen again	14 (4.3)
Observation	Follow closely the health status of animals	1 (0.3)
	Avoid close contact with sick animals	1 (0.3)
Medication	Regular vaccination	183 (56.1)
	Have drugs at home for emergency use	3 (0.9)
Community awareness		2 (0.6)
Consumption	Never consume sick animal products	2 (0.6)
Nothing can be done		7 (2.1)
**Categories**	**Challenges to outbreak prevention (N = 325)**	
No opinion		64 (19.7)
Medication	Lack of regular vaccination program/lack of vaccines	136 (41.8)
	Lack of available drugs in health centers and/or for animals	60 (18.5)
Service	Slow response during outbreaks	37 (11.4)
	No animal health service	26 (8.0)
	Governement has no protective mechanism against diseases	16 (4.9)
	Lack of disease surveillance	2 (0.6)
Fodder, climate	No feed provision by the Government during dry season	2 (0.6)
	Not enough good pastures in the area	1 (0.3)
	Drought	1 (0.3)
Disease awareness	Lack of community awareness	38 (11.7)
No challenges	The disease is not a problem	40 (12.3)
	Nothing can be done	1 (0.3)
	God protects the community, not the Government	1 (0.3)

## Discussion

The study showed that there were significant deficiencies in both the prevention and the control of zoonotic diseases in both pastoral regions. Pastoral respondents from the Afar and Somali region highlighted that Anthrax was among the top three diseases causing high mortality in their cattle and camel populations. The perceived significance of Anthrax among pastoral communities aligns with Anthrax being listed as one of the priority diseases by the Ethiopian Government [5]. Nearly half of the pastoralists who were familiar with zoonotic diseases identified Anthrax as the top zoonotic disease, followed by tuberculosis (32.2%), Foot and Mouth disease (8.2%), and rabies (6.7%). Brucellosis, on the other hand, that is also on the disease priority list of the Ethiopian Government, was not mentioned by any respondents as a disease of concern for them. However, previous studies have shown that brucellosis was endemic in Afar and SRS, circulating as well in livestock as in people [9].

A quarter (25%) of the interviewed households reported experiencing disease outbreaks in their areas within the last 12 months, including suspected cases of Anthrax. Overall, 59.2% of the households recalled experiencing livestock mortality as a result. These findings indicate that infectious diseases are consistently present and have a substantial negative impact on the health and economic of pastoral households in Afar and SRS. Anthrax is classified as an immediately notifiable disease, with even a single case considered an outbreak [12]. Interestingly, in a review conducted by Bahiru et al (2016) examining Anthrax cases between 2009 and 2013, only a few suspected animal cases were reported in pastoral regions, with no human cases documented [6]. This information contrasts sharply with the accounts provided by pastoralists and health care providers in our study, highlighting a discrepancy between the prior study’s findings and the current perceptions and experiences of the local communities. This raises the question whether Anthrax cases are highly under-reported in these pastoral areas. While sporadic outbreaks in both animals and humans are reported across the country [6,14–16], to our knowledge, there are no peer-reviewed published reports specifically addressing Anthrax in Afar and SRS. However, in addition to our study, there have been regular reports of suspected Anthrax outbreaks documented in grey literature and local news sources. For instance, in Afar, an outbreak affecting hundreds of people was reported in 2020 [11]. Similarly, in the SRS’s Jarar zone in 2021, one woman died and 39 individuals fell ill with suspected gastrointestinal Anthrax after consuming meat from an infected camel. The following year, in December 2022, another outbreak was reported in Kabribayah district (SRS), where two out of five people who consumed meat from an infected camel tested positive for Anthrax through PCR diagnostics (*personal communication Osman Y*, *Jigjiga One Health Initiative*). These anecdotal reports provide additional evidence of the presence and impact of Anthrax in these regions.

Zoonotic diseases pose a significant health threat to animals, pastoralists, and their livelihood. Raising awareness about these diseases is a crucial step in reducing the risk of transmission, particularly in areas where there is weak or no surveillance and control strategies in place, and where pastoralists have limited access to health care services.

Interestingly, more than half of the pastoralists (52.4%) were aware that animals could transmit diseases to people. This awareness level is higher compared to Tigray for instance, where over 60% of the community did not know Anthrax to be zoonotic [17].

Disease knowledge of Anthrax was higher among our pastoral respondents than the one observed in other highland regions [18]. It was also higher than the one reported from our interviewed health care providers. Pastoralists have long been recognized as a group with extensive knowledge about their animals, including the diseases that affect them [19]. Their close daily interactions with their animals fosters a strong bond and enables them to develop acute observation skills regarding animal health, and disease pattern. Moreover, pastoralists possess a wealth of traditional knowledge that has been orally transmitted across generations. This was also reflected in our study, with elders particularly playing the most important role in transmitting disease knowledge and not the formal education system. This accumulated knowledge is a valuable resource, providing them with insights and expertise in managing animal health within their unique environments. In addition, they gain hands-on experience during disease outbreaks within their own herds or through exposure during migrations. In our study, pastoralists who were migrating were significantly more knowledgeable about disease symptoms, transmission and their prevention than participants who were not migrating. The strong networking and community sharing practices commonly practiced among pastoralists facilitates the sharing of vital information.

Both animal health workers and community health workers are frontline health care providers for pastoral communities. Their unique position within the community makes them valuable contributors to disease control and prevention efforts, particularly if stronger joint animal-human surveillance systems are to be established. Although a third of pastoralists’ knowledge was provided by animal health workers, only few public health workers (4.1%) contributed to community awareness. A similar lack of involvement of HEWs in disease knowledge transfer was observed in a study conducted in Gondar [15]. Our study showed that also the role of the media as a tool to raise disease awareness was very limited in these areas.

While pastoralists generally possess a good understanding of diseases affecting their livestock, including mode of transmission and preventive measures, various external socio-economic and environmental factors impede their ability to mitigate risks, exposing them to pathogens such as Anthrax and Brucellosis. The great majority of respondents engaged in risky behavior such as consumption of sick animal raw products (milk, organ meat), conducted backyard slaughter also of sick animals and were using hides and wool of sick slaughtered animals. The consumption of recently deceased and sick animals has been reported as a widespread practice in Ethiopia [20–22]. Certain factors deeply ingrained in the pastoral cultural system and socio-economic fabric tend to outweigh the knowledge of disease risks. These factors include the lack of viable alternatives, the need for livelihood and basic survival in areas frequently affected by climatic shocks, conflicts, poverty, famine, among other challenges. Notably, a poor economic background has been observed as the primary driver behind farmers in Tigray, for instance, consuming meat from sick animals and selling the hides of dead animals, despite the community’s awareness of Anthrax [14]. Moreover, our study highlighted a stark difference in disease knowledge between male and female participants. Considering the daily role of pastoral women in the household including milking, cooking and marketing [23], it is of paramount importance to assess the sources of this inequality in disease knowledge gap in order to provide better health awareness and empower women to mitigate disease exposure risks.

When their animals fall ill, pastoralists primarily rely when they are at home, on self-administered medications (73.6%) or drugs provided by veterinarians (42.5%). Whereas, during migration, they use also traditional medicine (37.1%), besides self-administered medications. The use of plant-based treatments is an important aspect of the healthcare in Ethiopia [24]. Various plant species are used to treat Anthrax in the Highlands but also in Afar in particularly [24,25]. Likewise, over a quarter of the respondents in SRS indicated that they would turn to traditional medicine when family members in the household fell ill. The high percentage of self-administrated medications raises here important quality and safety concerns, such as sourcing of drugs, including black-market origins, pastoralists knowledge about drugs (effects, side-effects, dosage), appropriate disease diagnosis and the fostering of drug resistances.

During disease outbreaks, such as Anthrax for example, pastoralists typically take immediately certain actions. These include notifying the village leader, who subsequently contacts local authorities (43.2%), treating sick animals as mentioned above (40%), and separating sick animals from the healthy ones (23.2%). On the other hand, pastoralists reported that animal health staff primarily engage in vaccinating healthy animals (62.9%) and treating sick animals (36.3%). However, the study highlighted limitations in veterinary services regarding prevention and interventions efforts. One of the limitations was the lack of available drugs on the market and challenges in accessing veterinary facilities. A small number of respondents (19.2%) claimed that animal health workers would not take any action during outbreaks, although the study did not provide the underlying reasons for this observation. Others stated that the response time by animal health services was too slow. It is worth noting that Africa experiences some of the longest delays between outbreak detection, interventions and public communication [26,27].

Early reporting of diseases is essential for individual owner as it allows for prompt treatment, benefiting both the affected animals and the broader community by reducing potential spread to other herds [28]. Effective communication within the community, and across disciplines is essential as it can alleviate the burden on human health by providing awareness, reducing exposure, and implementing preventive measures. It also helps prevent the spread of diseases to the environment and wildlife populations. The majority of pastoralists (76%) were well informed and aware that vaccination protects their animals against diseases. However, less than half of them did vaccinate their animals in the last 12 months. This limited vaccination coverage can be attributed to the scarcity of vaccine services (from production to regional distribution) and logistical challenges (i.e. transport, storage, cold chain) [29]. In many cases, vaccination is only provided during disease outbreaks as part of a ring vaccination strategy, rather than being implemented as a routine preventive measure [29]. Our study findings aligns with this pattern, as over a third of the pastoralists reported vaccinating their livestock solely during the onset of outbreaks. The shortage of available vaccines and the absence of routine preventive vaccination emerged as the top challenge (41.8%) identified by pastoralists in preventing future outbreaks. Other limiting factors included the mistrust in vaccines, as expressed by 50 pastoralists (9.8%), who believed that vaccines could make animals sick, the lack of veterinary services providing vaccinations in certain areas (1.7%) and the insufficient knowledge among pastoralists regarding the importance of vaccines (7.9%).

Immediately upon the onset of an outbreak, pastoralists often move their animals to safer locations as a preventive measure. However, this practice can lead to delays or even cancellation in animal vaccination when animal health workers arrive at the settlements after the pastoralists have already relocated. These emergency animal movements whether conducted by individuals or the entire community, can potentially contribute to the spread of diseases to other areas and herds, including wildlife, as well as result in new contamination of pastures. Additionally, inadequate removal of infected carcasses, as observed in our study, and potential mixing of herds at watering points, for instance, further compound the risk of disease transmission.

The findings of this study highlight the unique and challenging nature of disease outbreak prevention and control in pastoral contexts. A transdisciplinary approach is crucial, wherein animal health workers integrate local knowledge and attitudes into their responses to outbreaks, taking into account animal movements and other contextual factors. By incorporating the perspectives and practices of pastoralists, more effective strategies can be developed to mitigate the impact of disease outbreaks in these communities.

The study showed deficiencies in public health service response to zoonotic diseases. Multiple challenges were identified, with a major limitation attributed to a lack of disease knowledge among health staff. The absence of laboratory confirmation and proper treatment protocols further exacerbate the problem, leading to a sense of mistrust among patients.

Specifically, 94.1% of the staff were unable to identify any symptoms of brucellosis, while 72.5% were unable to identify any symptoms of Anthrax. Similar gaps in professional knowledge regarding Anthrax and rabies were also observed among healthcare providers in Jimma [30].

None of the health technicians or Health Extension Workers (HEWs) were knowledgeable about the symptoms of both diseases. It is the role of HEWs to provide community awareness, do patient triage, and offer frontline services. Having a strong understanding of disease symptoms becomes particularly crucial when laboratory pathogen confirmation is unavailable as seen in our study.

Although early stages of inhalation or gastrointestinal Anthrax present often non-specific symptoms and are easy to misdiagnose [31], 95% of Anthrax cases manifest as cutaneous infections, characterized by distinct and recognizable skin lesions [31,32]. With prompt treatment, the prognosis for cutaneous Anthrax cases is typically positive.

Thirteen healthcare providers identified the lack of disease awareness among the community as the contributing factor to patients delaying seeking healthcare services. However, if HEWs are not knowledgeable about the diseases, they are unable to effectively communicate disease awareness to the communities. The study showed that a significant proportion (45.5%) of the pastoralist respondents were indeed unaware that Anthrax could be treated with medications in people.

Furthermore, the study showed an absence of clear protocol at health facility level for managing zoonotic diseases. One- third of the healthcare providers reported placing suspected Anthrax patients in quarantine, isolating them in a separate room within the health facility. This room was kept in complete darkness and any contacts with other individuals, including family members was strictly prohibited. This practice may contribute to patients feeling a sense of mistrust, anxiety, and discomfort, which in turn, can also lead to delays in seeking medical attention or even avoidance of healthcare facilities altogether

The strength of this study lies in the new knowledge of disease surveillance, prevention and control among pastoral communities living in remote areas that are sometimes difficult to access and for which data is lacking. The results of this study will support decision makers in tailoring disease prevention and response in those communities. However, the study has also limitations in terms of selection bias with few study sites included hence, the results may not be generalizable for the whole of both regions. We mitigated these biases by understanding the following: 1) all respondents belong to a similar group of people sharing socio-cultural, indigenous understanding and practices; 2) pastoralists of the entire regions were either migrating to the selected study sites with their animals during drought periods, or at least transiting through them;3) Similarly, the study sites encompass significant market areas that attract pastoralists from each region.

## Conclusion

Despite Anthrax being a notifiable disease, there is a significant disparity between the official records and the actual number of cases occurring within the pastoral communities. This discrepancy suggests that diseases such as Anthrax and brucellosis are likely to be significantly under-reported, misdiagnosed, and inadequately treated. Limited disease knowledge is one of the important bottleneck that the study identified.

A One Health approach in surveillance and intervention is warranted in those areas, where people and animal are so intrinsically linked. The integrated animal-human surveillance system that was implemented in SRS through the Jigjiga One Health Initiative (JOHI) could serve as an example. It demonstrates that joint zoonosis surveillance can lead to improved diagnostics and facilitate a more rapid and coordinated response compared to conventional surveillance strategies [33]. Both the media and the public health sector can play a significantly more prominent role in achieving better disease awareness, particularly targeting women pastoralists. Moreover, considering the extensive knowledge possessed by pastoralists, particularly regarding their animals and environment, interventions would greatly benefit from closer transdisciplinary collaboration with the community. Such collaboration would foster mutual trust, leading to improved notification and reporting of cases, as well as more rapid and widely accepted interventions. Leveraging the widespread availability of mobile phone networks and engaging key informants within the communities can greatly enhance tracking pastoralists’ movements. This, in turn, facilitates the implementation of timely and appropriate interventions, such as emergency ring vaccination during disease outbreaks. This approach empowers both the community and healthcare providers to work together more effectively, leading to improved disease prevention, surveillance, and response measures.

## Supporting information

S1 Data Questionnaire data from health staff on knowledge-attitude and practice towards brucellosis and Anthrax. (XLSX)

S2 Data Questionnaire data from pastoralists in Afar and Somali region on knowledge-attitude and practice towards zoonosis and brucellosis and Anthrax in particular. (XLSX)
